# Under‐Interpretation of Neuroimaging Data in Insanity Assessment: A Hidden Risk

**DOI:** 10.1002/bsl.70006

**Published:** 2025-07-02

**Authors:** Camilla Frangi, Alexa Schincariol, Pietro Pietrini, Giuseppe Sartori, Stefano Ferracuti, Cristina Scarpazza

**Affiliations:** ^1^ Department of General Psychology University of Padova Padova Italy; ^2^ Padova Neuroscience Center (PNC) University of Padova Padova Italy; ^3^ IMT School for Advanced Studies Lucca Lucca Italy; ^4^ Department of Human Neurosciences Sapienza Università di Roma Rome Italy; ^5^ IRCCS S Camillo Hospital Venezia Italy

**Keywords:** cognitive bias, criminal liability, expert witness, forensic psychiatry, insanity defence, law, neuroimaging, neuroscience

## Abstract

Neuroimaging data can provide valuable insights into insanity evaluations, but the debate over its use for legal purposes is far from resolved. While much attention has been given to the risks of over‐interpretation, potential errors stemming from under‐interpretation received less scrutiny. In this paper, we aim to showcase how this error may influence the results of an insanity evaluation by presenting an Italian forensic case. The defendant presented with intellectual disability and psychotic symptoms coupled with multiple brain abnormalities that were interpreted as variant of normal neuroanatomy. The case is discussed in detail. This article offers an insight into a neglected issue in forensic neuroscience, destined to gain prominence as this discipline becomes increasingly important in criminal justice systems worldwide. We recommend the use of a multidisciplinary approach to insanity to reduce the likelihood of error. In this context, neuroimaging can play an important role, and its interpretation should strictly adhere to guidelines to minimize the possibility of both over‐interpretation and under‐interpretation.

## 1 Introduction

Although neuroimaging techniques made their entry into the courtroom more than 4 decades ago, the controversy regarding their use for legal purposes is far from a settlement (Scarpazza, Zampieri, et al. 2021; Vitacco et al. 2021, 2024). The trial of John Hinckley Jr. in 1982, besides being among the most high‐profile trials in recent American history, was one of the first to witness neuroimaging among the trial's protagonists (Khoshbin and Khoshbin 2007). This case marked a significant milestone in the intersection of neuroscience and law, as it allowed neuroimaging to show its potential as a source of information about the connection between brain alterations and behaviour in the assessment of legal insanity. At the same time, this trial launched the debate between proponents and opponents to the use of such techniques in the legal arena (Bonnie et al. 2008). In this case the influence those scans had on the jury was probably driven by their novelty, rather than by the scientific relevance of the evidence itself. However, this case introduced the possibility to utilise brain scans as a supporting piece of evidence in insanity evaluations and launched the methodological debate on how they can be correctly applied. A similarly historic case in Italy was the one which ended in the famous ‘Como ruling’ (2011), in which the judge distanced themselves from the thesis of their own expert witness to embrace the one from the defence consultants. The latter claimed insanity for a woman who was accused of her sister's murder and supported their clinical forensic reasoning with structural imaging data and the analysis of genetic polymorphisms (Farisco and Petrini 2014; Turone 2011). This case became particularly famous in Italy for the innovative methodology applied by the defence consultants, but above all for how the judge justified their ruling, stating: ‘The conclusion of the defence is much more convincing than the conclusion of the expert witness for the judge. The logical reasoning and argumentations of the defence appear to be very convincing as they rely on a solid and very complete methodology that ensures a very in‐depth examination of the defendant as the consultants used the classical clinical interview, structured interviews, psychopathological tests, neuropsychological tests and, to complete the assessment, a structural brain scan of the defendant’.

From these cases it is clear that the use of neuroimaging evidence could be both virtuous and harmful, depending on the way these techniques are applied. In the following paragraphs, we will highlight the problems of classical insanity evaluation, the potential relevance of neuroimaging, the criticisms towards its use, and how to mitigate these criticisms. While opponents of the introduction of neuroimaging in courts are worried about the possible over‐interpretation of imaging results, the risk of the opposite, namely the under‐interpretation of neuroimaging results, is a real problem as well. Both concerns are critical to point out and avoid, although the latter is less discussed in the literature. Before delving into this topic, it is of the utmost importance to summarize the state of the art regarding this controversy.

### 1.1 The Problems of Classical Insanity Evaluations

Most legal jurisdictions incorporate provisions in their criminal legislation to safeguard individuals whose unlawful actions stem from mental incapacitation (Adjorlolo et al. 2019). One such provision is the insanity defence, which upholds that individuals who commit heinous acts, such as murder, under the influence of a mental illness, should not bear criminal responsibility due to their lack of requisite mental capacity or intent (Desmarais et al. 2008). Although specifics may change according to the country's legislature, an evaluation of legal insanity in Western countries typically revolves around three key inquiries: (1) the presence of an altered state of mind that impairs or abolish the ability to understand and to will; (2) the alteration of this state of mind at the time of the crime commission; and (3) the causal relationship between the altered state of mind and the crime. An over‐simplified example would be the one of pyromania: in a case of homicide, this disorder could be relevant if the victim died in a fire but not if they were stabbed (Scarpazza et al. 2021). The expression ‘causal link between crime and altered state of mind’ does not refer to liner causality between the two (i.e., that the altered state of mind caused the crime), but rather to the contribution of the altered state mind to the factors causing the crime. This view stems from a specific model of causation, the so‐called INUS (*Insufficient but Necessary part of an Unnecessary but Sufficient condition*) model, which deems an event as being caused by several insufficient causes whose combination becomes sufficient for the event to occur (Anckarsäter et al. 2009; Mackie 1974). However, several issues may complicate the examination of these inquiries.

First, although insanity is a juridical construct, expert witnesses are often clinicians who are asked to determine whether a defendant was insane when they committed a specific crime. To allow such an evaluation, the juridical construct of insanity needs to be translated into a clinical one, that is, that symptoms and/or diagnoses than can potentially impair the state of mind of a person need to be identified and a degree of impairment which excludes criminal responsibility should be established. This translation process is continuously evolving with the law: if the legal criteria for criminal responsibility or the evidence acceptable to argue insanity change, the clinical criteria used to assess insanity should change accordingly. Similarly, these clinical criteria should always consider the latest scientific findings on relevant themes and incorporate the recommendations developed by researchers. This is true of any kind of scientific evidence applied to legal settings: a specific kind of evidence should be used only when it is deemed acceptable by the legal system (be it through a specific law or a judge confirming its acceptability).

Second, criminal behaviour has a complex and multifaceted structure, which is built on a combination of social, psychological and biological factors (Barnes et al. 2022; Raine 2008). Notably, a growing body of research indicates that neurobiological factors, including genetics, epigenetics, and both structural and functional neural correlates, play a significant role in influencing criminal behaviour (Anderson and Kiehl 2020; Darby, Horn, et al. 2018; Darby, Joutsa, et al. 2018; Fumagalli and Priori 2012; Iofrida et al. 2014; Palumbo et al. 2018; Pietrini et al. 2000; Rigoni et al. 2010; Romero‐Martínez et al. 2019; Sajous‐Turner et al. 2020; Schug et al. 2010).

Third, most insanity evaluations concern individuals with a phenomenological manifestation potentially compatible with a psychopathological diagnosis or a mental disorder. Only in a minority of cases the evaluation pertains a person probably suffering from a neurological disorder. Psychopathological disorders lack biomarkers (Perlis 2011; Prata et al. 2014), which are biological data supporting a clinical status. Biomarkers are present for most medical problem (e.g., the diagnosis of multiple sclerosis is corroborated by the presence of oligoclonal bands in the cerebrospinal fluid). Nothing similar is available for psychopathological disorders, with the consequence that psychopathology suffers from low inter‐rater reliability, meaning that different clinicians observing the same patients are likely to reach different diagnostic conclusions (Aboraya et al. 2006; Chmielewski et al. 2015; Miller 2001; Miller et al. 2001; Regier et al. 2013). Similarly, the inter‐rater reliability of insanity evaluation is low (Guarnera and Murrie 2017), even in jurisdictions with three independent experts summoned by the judge (Gowensmith et al. 2013).

Fourth, as Dror and colleagues have thoroughly illustrated (Dror 2018; Dror et al. 2021; Dror and Murrie 2018), all forensic sciences, including psychology and psychiatry, are by no means immune to the presence of cognitive biases, defined as unconscious distortions of thinking and reasoning. The Human Expert Performance model (HEP; Dror 2016) comprehensively describes the influence of cognitive biases in forensic reasoning across all disciplines. Its key concept is that biases can emerge both at the level of observations (i.e., the actual observation of the data) and at the level of the conclusion (i.e., how the observed data are interpreted to reach a forensic conclusion; Dror 2016). In forensic psychopathology, different experts can report different symptoms (level of the observations), or they can observe the same symptoms and interpret them differently, leading to different diagnostic conclusions (Dror and Murrie 2018). An example of biases at the level of the observation lies in a case described by Scarpazza et al. (2021): starting from the same clinical interview, the defendant was described as free of any relevant symptoms by one consultant and as presenting with paranoid and bizarre delusions by another expert. Clearly, the two consultants reached completely opposite conclusions on the diagnosis and consequently on insanity. An example of bias at the level of the conclusion is the case described by Melle (2013), in which the defendant's symptoms were consistently described by different clinicians but were given discrepant interpretations (bizarre vs. not bizarre delusions), leading to different diagnoses with a distinct impact on insanity.

Moreover, preliminary evidence suggests that in forensic psychopathology biases are mainly present at the level of the observations (i.e., different experts observe different symptoms; Scarpazza and Ghidini 2023). This hints at biases being linked to the intrinsic complexity of psychopathology rather that adversarial dynamics linked to the legal context (i.e., the so‐called *adversarial allegiance*, which refers to the tendency of forensic experts to align their opinion to the one that benefits the party they usually work for the most; Guarnera et al. 2017; Murrie et al. 2013).

Notably, as insanity evaluations are considered scientific evidence, they should comply with the Daubert criteria and help judges reach a decision ‘beyond any reasonable doubt’. It is therefore abundantly clear that the intrinsic uncertainty of forensic psychopathology needs to be reduced and the inter‐rater reliability between experts needs to be increased.

### 1.2 A Suggestion for Mitigating the Problem of Low Inter‐Rater Reliability

To mitigate the complexity of legal responsibility, overcome the subjectivity of diagnosis, increase concordance among experts, and reduce the problem of cognitive biases, an integrated and multidisciplinary evaluation system has been recently proposed (Scarpazza, Miolla, et al. 2021; Scarpazza, Zampieri, et al. 2021). This approach involves a comprehensive assessment of the defendant by means of both qualitative and quantitative techniques. Unstructured clinical interviews (often considered in insanity evaluations) should be therefore combined with structured clinical interviews, psychopathological and neuropsychological testing, and with measures investigating possible neurobiological alterations, when appropriate.

This approach is founded on a simple logical assumption: since the error rate of classical insanity evaluations is too high to reach a conclusion ‘beyond any reasonable doubt’ due to their low inter‐rater reliability, this kind of evaluation by itself is not sufficient to answer to questions posed by a judge. On the contrary, if we combine the results coming from different disciplines (psychiatry, neuropsychology, psychopathology, neuroscience), each result will have an intrinsic error rate, but if they converge in supporting one hypothesis, it will be very difficult to support the opposite hypothesis without falling into cognitive biases.

The aim of the multidisciplinary approach is to strengthen the scientific foundations of insanity evaluations. Since science is based on falsifiability and verifiability, evaluations should be rooted in methods that allow falsification. Formulating and testing a hypothesis with the same methods can potentially lead to confirmation bias, therefore it is useful to use methodologies from different disciplines to test the robustness of a scientific hypothesis, which is deemed valid if it accounts for all available scientific evidence.

In this framework, the use of neuroimaging is not always needed, but sometimes it might contribute to disentangling clinical uncertainties (Sartori et al. 2016; Scarpazza, Zampieri, et al. 2021). Structural neuroimaging techniques may thus help fulfil the need for an integrated, multidimensional assessment of the defendant, offering a quantitative measure of the degree of brain alteration. Such evidence can be useful in disambiguating the presence or absence of pathology and is not subject to the phenomena of exaggeration or faking of symptoms by the subject (i.e., *malingering*; Adetunji et al. 2006; Gorman 1982). When expert witnesses agree on the symptoms and/or diagnosis of the defendant, neuroimaging is not needed. The problem is, this level of agreement between experts is extremely rare, at least in Italy.

The HEP model (Dror 2016; Dror and Murrie 2018) gives a simple framework to clarify these concepts. In insanity evaluations, expert witnesses can disagree: (i) on reported/observed symptoms (inter‐rater reliability at the level of the observations); (ii) on how symptoms are interpreted to reach a diagnosis (inter‐rater reliability at the level of the conclusion); (iii) on the impact that the diagnosis/symptoms have on the commission of the crime (inter‐rater reliability at the level of the second conclusion). In forensic psychopathology disagreement often arises at all levels of the evaluation: experts disagree on symptom description, on the diagnosis, and, most importantly, on the impact of the altered state of mind on the commission of the crime. Neuroimaging can contribute to disentangle clinically ambiguous cases, allowing the experts to focus their discussion on the relevance of the mental disorder to the crime. For a case description see Scarpazza, Zampieri, et al. (2021).

### 1.3 Critiques to the Introduction of Neuroimaging in Court

Opponents of neuroimaging in insanity defences (Farisco and Petrini 2014; Husted et al. 2008; Martell 2009; Vitacco et al. 2020, 2021, 2024) raised numerous concerns regarding its application in courtrooms. A detailed discussion of these critiques has already been published elsewhere (Scarpazza et al. 2021).

First, they argue that neuroimaging fails to add any significant value to already existing information, contending a specific brain imaging finding is often associated with various diagnoses relevant to forensic cases. Second, they highlight the dynamic nature of the brain and neurological processes, cautioning against oversimplifying complex behavioural phenomena by relying solely on neuroimaging. Moreover, they criticise the tendency of neuroimaging experts to directly link brain data to criminal actions (Morse 2016; Perlin 2009). They also report that numerous scientists and professionals have questioned the admissibility of neuroimaging evidence based on the Daubert criteria,^1^ which includes testability, error rates, peer review, and scientific acceptance (Felthous and Saß 2008; Moriarty 2008). Furthermore, they point out a lack of published studies validating the efficacy of brain imaging in identifying the functional deficits necessary for a successful *not guilty by reason of insanity* (NGRI) verdict, such as the inability to discern right from wrong. Finally, they assert that current neuroimaging techniques lack the sophistication required to retrospectively evaluate mental states. The timeframe of this debate highlights how the discussion into how and to which extent neuroimaging should be used in court is still ongoing, with researchers failing to find an agreement on the subject.

Among the various criticisms against the use of neuroimaging in the assessment of criminal liability, the concern of the *reverse inference* is particularly prevalent in both the legal and mental health community (Morse 2016; Vitacco et al. 2020). The logical fallacy named *reverse inference* occurs when one assumes that the existence of a specific cognitive process is implied by the presence of neural activity in a particular brain area, or when a pathological behaviour is inferred solely by the identification of a change in the brain structure (Poldrack 2006; Scarpazza, Pellegrini, et al. 2018). According to Perlin (2009), neuroimaging experts frequently fall prey to this fallacy, asserting a direct correlation between criminal behaviour and brain data and claiming this link is relevant for an insanity defence. Such an approach thus consists in an over‐interpretation of the neuroimaging data.

An eloquent example of over‐interpretation of neuroimaging data is found in the famous US criminal case involving Herbert Weinstein^2^ (Davis 2017). Weinstein, a 65‐years old man, strangled his wife and then threw her from the 12th floor of a Manhattan building. The defence strategy relied on the opinion of a neurologist who identified an arachnoid cyst through brain magnetic resonance imaging and directly linked this brain anomaly to the criminal act (Choi 2017; Rojas‐Burke 1993). In this regard, although literature provides evidence of associations between the presence of arachnoid cysts and the development of psychotic symptoms (Ferracuti et al. 1991), no one tested Weinstein's inhibitory abilities or moral reasoning, nor tested for the presence of psychosis in the defendant. Additionally, the fact that the defendant had never previously shown violent behaviour towards others, despite the presence of the cyst, was not considered. Weinstein's defence, therefore, relied almost exclusively on the use of reverse reasoning, eliciting sharp criticism from the international neuroscience community and unsettling both supporters and opponents of neuroimaging in the courtroom (Bigenwald and Chambon 2019). A similar case in Italy involved a serial killer, Gianfranco Stevanin, accused of murdering nine women during extreme sex acts. The defendant was involved of a car accident in adolescence, leaving him with a lesion to the frontal lobe. In the first‐degree trial the defence strategy was centred on this lesion, with the lawyer claiming insanity due to dis‐inhibition: the man began the sexual acts with his victim's consent, but when he realized the woman was suffocating, he was not able to inhibit his sexual impulse due to the brain lesion. The defendant was initially recognized as insane. During the appeal, the prosecutor demonstrated that Stevanin had the very same extreme sexual intercourse with his girlfriend but always managed to stop before killing her. This is an incontrovertible demonstration that he was able to inhibit his sexual impulses if he wanted to, and in the end, he was recognized as sane and sentenced to life in prison for multiple first‐degree murders.

These two cases clearly highlight that brain imaging needs to be properly weighted and integrated with other sources of evidence to be used in forensic settings. Otherwise, its use is improper and harmful to the juridical process.

### 1.4 The Problem of Over‐Interpretation of Neuroimaging and a Way to Mitigate It

The risk of over‐interpretation is probably one key factor in the opposition to the introduction of neuroimaging in court, as many examples of this phenomenon are present in the literature. Recognizing this risk and recognizing the charming nature of brain images (Aspinwall et al. 2012; Fuss et al. 2015; McCabe and Castel 2008; Schweitzer et al. 2011; Weisberg et al. 2008), the literature has recently witnessed the introduction of guidelines created to guide the interpretation of structural neuroimaging findings in forensic settings, with the aim of mitigating, and possibly erasing, the risk of over‐interpretation (Scarpazza et al. 2018).

**FIGURE 1:**
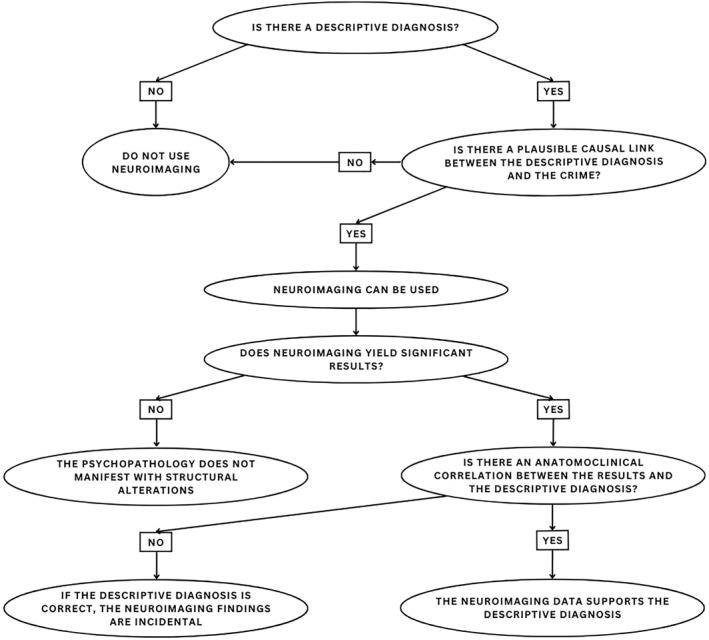
Graphical summary of the guidelines for the interpretation of structural neuroimaging in forensic settings (adapted from Scarpazza et al. (2018)).

These guidelines stem from four principles specifically created to avoid cognitive biases: (1) neuroimaging results should be coupled with behavioural findings to avoid false positives; (2) the criminal behaviour cannot be considered a symptom to avoid circular reasoning; (3) not every brain abnormality leads to behavioural symptoms, necessary to avoid determinism and confirmatory logic; (4) do not infer the presence of a pathology from the presence of brain abnormality to avoid reverse inference. In Figure 1 the flowchart guiding the application of the guidelines to forensic cases can be appreciated.

First, according to rule 1, the defendant should manifest behavioural symptoms (or have a clinical diagnosis). This is fundamental because in the absence of any clinical manifestation it would be impossible to provide a reliable interpretation of any neuroimaging results. If the defendant does not manifest any symptoms, neuroimaging should not in any way be used. If the defendant manifests symptoms, it is possible (though not mandatory) to use neuroimaging. In criminal trials expert witnesses often disagree on symptom description and diagnosis (Guarnera et al. 2017; Guarnera and Murrie 2017; Melle 2013; Scarpazza and Ghidini 2023), and neuroimaging can help disentangle clinically ambiguous situations. In a recent murder case followed by the authors, the expert witness for the judge diagnosed the defendant with depression, whereas the defence consultants diagnosed them with chronic alcoholism (based on blood analyses and cognitive testing). Due to the influence of excessive alcohol consumption on the brain, a brain scan was requested, which highlighted the presence of atrophy which was highly likely to be linked to chronic alcoholism, thus supporting the diagnosis of the defence.

Second, the guidelines require that the identified descriptive diagnosis is in causal link with the crime. Although the wording ‘causal link’ might seem excessive, it does not refer to a causal relationship between the symptoms/diagnosis and the crime. Indeed, criminal behaviour is a complex phenomenon and cannot be explained deterministically by psychiatric, behavioural, or cognitive symptoms alone (Barnes et al. 2022; Raine 2008). The wording used in the guidelines, however, is reminiscent of one of the principles of forensic psychopathology, namely the necessity of a link between a psychopathological manifestation and a criminal behaviour for said manifestation to be relevant for insanity. It is not sufficient to demonstrate the presence of relevant symptoms, but it is also necessary to explain how the symptoms are linked to the crime. The causality mentioned here, therefore, identifies a contribution that the psychopathological symptoms might have had in the initiation of a specific criminal behaviour, rather than pure causality (as in symptoms fully causing the crime).

Third, it is possible to apply neuroimaging only if symptoms that may explain the commission of the crime are present. For results to be relevant in forensic settings, they need to be in anatomo‐clinical correlation with the symptoms manifested by the defendant. If the anatomo‐clinical correlation is not present, neuroimaging results need to be considered incidental and cannot be used to support the presence of any pathology. It is the case, for instance, of a woman prosecuted for murder, who was diagnosed with dependent personality disorder and manifested high impulsivity at the formal neuropsychological tests. Imaging results revealed a decreased volume of the occipital cortex. In this case, even if significant results emerged, they are irrelevant to corroborate the clinical symptoms of the defendant as an anatomo‐clinical correlation is missing (for the sake of clarity: it is not possible to say that the symptoms are not real because they do not have a brain correlate. This reasoning would reflect the reverse inference logical fallacy).

As it is clear from these guidelines, the criteria to evaluate insanity remain rooted in the clinical profile of the defendant (Murrie et al. 2013), but brain scans can offer objective data to support clinical diagnoses in cases where experts disagree (Kassin et al. 2013; Sartori et al. 2011). Neuroimaging exams are not any different from other medical exams that may be available in the defendant's medical record. At times their use may reveal undiagnosed neurological problems which explain behaviour of a person, or of a criminal defendant. An example of this may be found in a forensic case already published in the literature (Sartori et al. 2016), which reports the insanity evaluation of a man charged for paedophilia who also manifested severely impulsive behaviours in his daily life. The expert witnesses for the judge diagnosed him with idiopathic paedophilia and therefore deemed him sane at the time of the crime, as in Italy paedophilia is not relevant for insanity due to the principle of *actio libera in causa*, that is, if an individual committed a crime while incapacitated but they caused said incapacitation knowing it might lead to a crime, they are held responsible for the crime. The defence consultants requested a brain MRI as they suspected the defendant might suffer from the behavioural variant of fronto‐temporal dementia. The brain scan revealed the presence of a clival chordoma, a slow growing tumour of the notochord which was displacing the hypothalamus and pituitary gland and compressing the frontal lobe. After the tumour was removed, all symptoms (including paedophilic urges) disappeared. Sometimes neurological disorders present with bizarre behavioural symptoms and are mistakenly diagnosed as psychiatric disturbances (Butler and Zeman 2005; Keshavan and Kaneko 2013; Lyketsos et al. 2007). In this case, the neuroimaging results were dismissed by the expert witnesses for the judge without even considering whether the results could be of use in the evaluation. For a more detailed discussion of this case, see Farisco and Petrini (2014) and Scarpazza et al. (2018).

While instances of over‐interpretation of neuroimages are now well recognized, courtrooms also witness the opposite error, namely the under‐interpretation of brain imaging data. As will be outlined in this article, this error involves underestimating evidence of structural brain alteration that proves a direct anatomo‐clinical correlation with the descriptive diagnosis. This under‐interpretation error, although much less considered than its counterpart, proves particularly detrimental and dangerous both from a legal and a clinical perspective: on one hand, there is the risk of incarcerating an individual who have a brain pathology that negatively impacted on their capacity to understand and to will at the time of the offence; on the other hand, there is the denial of necessary medical care and the further risk of worsening the individual's condition due to the prison environment.

## 2 The Legal Framework of This Paper

### 2.1 The Concept of Legal Insanity in the Italian Penal Code

Before detailing the case to be presented in this paper, it is fundamental to briefly introduce how insanity is evaluated in the Italian justice system, as not all readers may be familiar with it. The key concept in insanity evaluation is accountability (*imputabilità*), in that only people who are criminally responsible for their actions can be prosecuted. Accountability is defined as the capacity to understand and to will (*capacità di intendere e di volere*), which can be roughly described as the capacity to grasp the moral and legal disvalue of one's actions and the capacity to change one's behaviour to avoid committing a crime.^3^ According to the Italian Penal Code, a person is not criminally responsible for a crime if they were in a ‘mental state’ (with the exact words used in the Penal Code) which abolished their capacity to understand and/or to will due to illness. This is what is known as full insanity (*vizio totale di mente*).^4^ Additionally, a person is partly criminally responsible for a crime if they were in a mental state which greatly diminished (without abolishing) their capacity to understand and/or to will due to illness. In this case, the person does not receive an NGRI verdict but rather a diminished sentence due to partial insanity (*vizio parziale di mente*).^5^ The Italian Penal code therefore tasks experts with the evaluation of the state of mind of the defendant to understand whether their ability to understand and to will were abolished or diminished due to an illness when the crime was committed. Although the discipline is starting to emphasise the importance of symptoms over a formal diagnosis (Løvgren et al. 2020), in most (but not all) cases, the legally relevant illnesses can be conceptualized as formal diagnosis of both neurological and psychiatric/psychopathological disorders. As a matter of fact, in written reports from insanity evaluations a formal nosographic diagnosis is always present.

### 2.2 The Expert Witnesses Involved in Insanity Evaluations in Italy

The Italian criminal justice system is mainly adversarial. During a trial, an insanity evaluation may be requested by any one of the parties involved (e.g., defence, prosecution), but the request must be approved by the judge. Once the evaluation is requested by the judge, all parties involved can appoint their own expert witness.

The expert appointed by the judge is called *Perito* and is considered impartial (i.e., free from adversarial dynamics). The judge may also appoint a team of experts, usually with a different background (e.g., a psychiatrist, a neuropsychologist, and a neurologist), who are asked to perform a joint evaluation of the defendant (i.e., they work together and turn in a joint report). The other parties are then allowed (although not required) to appoint their own experts, who can be at most the same number as the ones appointed by the judge.

The insanity evaluation is conducted by the *Perito*, while the experts appointed by the parties can only attend the evaluation. They are allowed to suggest additional testing and/or further examinations but are prohibited from meeting with the defendant without the *Perito* and the experts appointed by the other parties. This organisation ensures that all experts involved rely on the same information in their evaluation of the specific case.

### 2.3 Scientific Evidence in Court

In the Italian justice system, insanity evaluations are considered scientific evidence and therefore need to be in compliance with the so‐called ‘Cozzini criteria’ (Cass. Pen. Sez IV. N. 43786, of 13.12.2010), which are the Italian version of the Daubert criteria. They apply the same principles established by the U.S. Supreme Court, although in a different formulation. To be acceptable in court a scientific technique must be generally accepted in the scientific community; it must have been subjected to peer review and published; the evidence must have been independently tested; and the error rate for the technique must be known and acceptable. The Italian Penal Procedure does not have formal rules to balance probative value of scientific evidence against its prejudicial potential, but any scientific evidence submitted is evaluated directly by the judge, who can either accept or reject it based on its reliability, relevance, and fairness.

### 2.4 The Strength of Scientific Evidence in the Italian Court

Importantly, the Italian jurisprudence applies the principle of ‘beyond any reasonable doubt’ to insanity evaluations: according to the ‘Palleschi ruling’ by the Italian Supreme Court of Cassation (Cass Pen. Num. 11897/2019 of 18.03.2919), the principle of ‘*in dubio pro reo*’ should be applied to insanity evaluations as well. In other words, the burden of proof is on the prosecution, who should demonstrate ‘beyond any reasonable doubt’ that the defendant is sane. This means that for a defendant to be declared sane, the judge must be convinced beyond any reasonable doubt of their sanity. For this reason, it is of the utmost importance that expert witnesses take into consideration all available medical data and rule out beyond any reasonable doubt their influence on the mental state of the defendant.

## 3 Aim of the Current Paper: The Problem of Under‐Interpretation of Neuroimaging Data

### 3.1 General Aim

This paper aims to draw attention to the under‐interpretation of structural neuroimaging data in forensic settings, an issue that has not been considered in the scientific literature. This manuscript will hopefully set the basis to open a constructive conversation on the scientific relevance of this issue and to provide some preliminary suggestions on how to mitigate this problem. To do so, we will describe one Italian criminal case. To facilitate its description, we will apply the guidelines for interpretation of structural imaging data in insanity evaluations (Scarpazza et al. 2018) and we will identify the level of disagreement between experts according to the HEP model (Dror 2016; Dror and Murrie 2018). Of note, the aim is not to provide empirical data supporting the efficacy of the guidelines for interpreting neuroimaging data, but to use them to describe the case highlighting the logical fallacies which may have affected the conclusion reached by the experts involved.

### 3.2 Neuroimaging in the Case Described

If neuroimaging techniques are applied in a criminal trial, their use usually aims at identifying any brain alterations in the defendant, which could be linked to observed behavioural disturbances. Depending on their size, alterations may be identified on a scan either by visual inspection (gross alterations; e.g., a tumour, atrophy in Alzheimer's disease) or by analysing the data with specific statistical techniques (subtle alterations, identified by using Voxel‐Based Morphometry; Scarpazza et al. 2013; Scarpazza and De Simone 2016). Some of the previously discussed critiques to the use of neuroimaging in court refer only to the results obtained after statistical analyses, in particular the following: (i) the brain is dynamic in nature; (ii) the inference group to individual cannot be made; (iii) neuroimaging do not comply with the Daubert standard (Vitacco et al. 2020). The use of brain magnetic resonance imaging is now routinely applied and decisions about the relevance of alterations in single individuals are performed on a daily basis in clinical practice.

Of note, the brain alterations that will be discussed in the case below were already evident at visual inspection, thus reducing the impact of the limitations of neuroimaging techniques when applied to single cases.

## 4 Case Description and Discussion

### 4.1 A Case of Double Homicide

The defendant was charged with the murders of his wife and his mother‐in‐law. He stated that voices inside his head saying ‘The prey is running away’ drove him to smother his wife. Right after he killed her, he tried to resuscitate her. Regarding the murder of his mother‐in‐law, he claimed he did it because the victim would not have been able to live without her own daughter. After the facts, the defendant called emergency services and confessed to the crimes right away. The judge asked for an insanity evaluation and appointed a psychiatrist, a neuropsychologist, and a neuroradiologist as expert witnesses (joint evaluation). The defence called a psychiatrist and a neuropsychologist as expert witnesses.

#### 4.1.1 The Insanity Evaluation

The defendant was a 55‐years‐old man with a history of obesity, hearing loss, and tinnitus. At the time of the forensic evaluation, he did not show any clear psychotic symptoms and appeared avoidant. During the clinical interview, he showed trivial concerns regarding his incarceration (e.g., absence of a diet catered to his health problems, sharing his cell with criminals), but did not seem to realise the legal consequences of his actions. He repeatedly complained about his daily problems, showing obsessive and perseverative tendencies on specific topics (e.g., his health problems, his unemployment).

A thorough neuropsychological evaluation of the defendant was conducted. During the evaluation he showed a depressive mood, tiredness and anxiety and he often interrupted the assessment to talk about something else, especially his health problems. The results revealed a mild cognitive disability (IQ = 50, as measured by means of the Wechsler Adult Intelligence Scale; Wechsler 2008) and impaired verbal comprehension, working memory, attention, verbal fluency, social cognition, and memory. Therefore, he showed a severe pattern of deficits in frontal functions and in social cognition. The neuropsychological assessment also included a test for malingering (Smith and Burger 1997; Van Impelen et al. 2014), which yielded positive results for exaggeration and/or faking of symptoms. Results of the cognitive evaluation are reported in Table 1.

**TABLE 1:** Results of the neuropsychological evaluation of the defendant.

Test name	Defendant's score	Cut‐off/population average (%)	Interpretation
Wechsler Adult Intelligence Scales‐IV (IQ)	50	100	Mild cognitive disability
Verbal Comprehension Index	76	100	
Perceptual Reasoning Index	58	100	
Working Memory Index	55	100	
Processing Speed Index	50	100	
Mini‐Mental State Examination	20	≥ 24	Impaired
Attentional matrices	8.5	≥ 31	Impaired
Switching verbal fluency	10.5	≥ 13	Impaired
Raven coloured progressive matrices	32.5	≥ 18	Normal
Boston naming test	19	≥ 43	Impaired
Phonemic verbal fluency	7	≥ 18	Impaired
Semantic verbal fluency	16	≥ 28	Impaired
Direct digit span	3	≥ 4	Impaired
Inverse digit span	0	≥ 2.5	Impaired
Direct corsi span	2	≥ 3.5	Impaired
Inverse corsi span	0	≥ 3	Impaired
Recall of a short story	4	≥ 8	Impaired
Test of constructive apraxia	10	≥ 8	Lower limit of normal
Clock drawing test	13	≥ 6.5	Normal
Frontal assessment battery	5	≥ 13.5	Impaired
Short verbal analogies test	14.5	≤ 14	Lower limit of normal
Test of emotion recognition			
Sadness	1	≥ 6	Impaired
Fear	10	≥ 8	Normal
Embarrassment	4	≥ 8	Impaired
Disgust	1	≥ 2	Impaired
Happiness	9	≥ 10	Impaired
Anger	3	≥ 6	Impaired
Envy	0	≥ 1	Impaired
Test of moral versus legal violations			
Moral behaviours—Not allowed	6	≥ 6	Normal
Moral behaviours—Severity	60	≥ 39	Normal
Conventional behaviours—Not allowed	6	≥ 5	Normal
Conventional behaviours—Severity	60	≥ 20	Normal
Rey 15‐item test	5	≥ 9	Positive for malingering
Structured inventory of memory malingering	51	≤ 14	Positive for malingering

**FIGURE 2:**
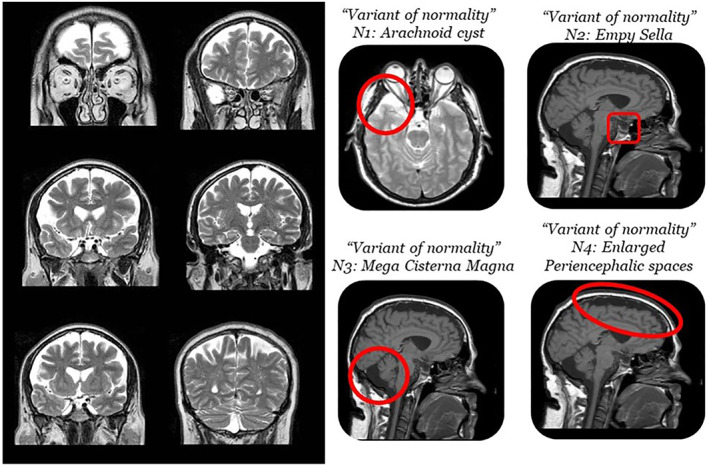
Brain abnormalities detected in the defendant. Images are provided in coronal (T2‐weighted on the left side of the image), axial (T2‐weighted, first ‘variant of normality’) and sagittal (T1‐weighted, variants of normality number 2, 3, 4).

The expert neuroradiologist ordered a magnetic resonance imaging (MRI) scan of the defendant's brain. The presence of enlarged peri‐encephalic spaces in different areas, especially around the frontal and parietal lobes, an arachnoid cyst, a mega‐cisterna magna, and an empty sella were identified (Figure 2).

#### 4.1.2 Conclusion of the Expert Witnesses Appointed by the Judge

The expert witnesses appointed by the judge concluded that the defendant was malingering all his symptoms, both psychiatric and neuropsychological, and therefore declared him completely able to understand and to will at the time of the crime. They then concluded that the brain was normal, as they considered these elements as variants of normality with no clinical correlates, a condition that can be observed in 10%–15% of the healthy population, based on a paper describing incidental findings on brain MRI (Vernooij et al. 2007). In other words, the psychiatric symptoms (hearing voices), the low IQ and other cognitive difficulties were deemed malingered, and the brain abnormalities were considered ‘normal variants in neuroanatomy’.

#### 4.1.3 Conclusion of the Expert Witnesses Appointed by the Defence

The defence consultants reached a different conclusion. They did not find enough evidence to exclude a first psychotic episode presenting with auditory hallucinations. The experts appointed by the judge questioned the presence of an intellectual disability due to two positive tests for malingering. However, the validity of such tests in people with intellectual disabilities has been questioned numerous times, with researchers warranting caution when using in forensic settings (Rogers et al. 2024; Vitacco et al. 2023). The literature suggests that the use of this kind of tests in patients with an IQ lower than 70 and patients with psychiatric disorders (which were both likely present in the defendant) leads to an inflation of false positives (Hays et al. 2000; Love et al. 2014; Van Impelen et al. 2014). One of the tests administered to the defendant was the Structured Inventory of Malingered Symptomatology (SIMS; Smith and Burger 2013), whose manual advises against the use with people with cognitive disabilities due to higher false positive rates. To guarantee the validity of these tests in this population, researchers suggest integrating their results with external evidence (Love et al. 2014; Van Impelen et al. 2014). In this specific case, the presence of the cognitive disability was supported by the clinical evidence gathered in the evaluation (e.g., tangential speech, difficulties understanding absurdities or abstract concepts). The defence consultant therefore concluded that the evidence gathered in the evaluation was not enough to support beyond any reasonable doubt that the defendant was malingering his psychopathology.

Concerning the brain imaging data, the defence consultants deemed it impossible to exclude beyond any reasonable doubt that the brain abnormalities identified were associated with the symptoms manifested by the defendant. As a matter of fact, these symptoms could be linked to the brain alterations, of which the defendant was not aware. The defence experts managed to provide a detailed analysis of how the defendant's psychopathological and neuropsychological deficits were consistent with the evidence revealed by the brain MRI.

First, the arachnoid cyst was located in the left temporal pole. This kind of abnormality has been repeatedly associated with psychiatric symptoms (Bahk et al. 2002; Da Silva et al. 2007; Mironov et al. 2014), which are then remitted upon removal of the cyst (Baquero et al. 2014; Vakis et al. 2006). The reported remission after removal indicates that the cyst most likely caused the psychiatric symptoms. Moreover, arachnoid cysts are associated with cognitive deficits in numerous domains, including visuo‐spatial memory and other visuo‐spatial functions, executive functions, verbal perception and memory, complex verbal tasks and visual attention (Agopian‐Dahlenmark et al. 2020; Gjerde et al. 2013; Gundersen et al. 2007; Wester 2008). These cognitive deficits are all present in the defendant. In addition, the mega cisterna magna has been repeatedly shown to be associated with both psychiatric and cognitive symptoms as well (Ferentinos et al. 2007; Pandurangi et al. 2014; Papazisis et al. 2007; Turan et al. 2010; Yazici et al. 2022). Given the close correspondence between the arachnoid cyst, the mega cisterna magna, and the psychiatric and cognitive symptoms, the defence consultants concluded that it was impossible to support beyond any reasonable doubt that the cyst and the mega cisterna magna were asymptomatic.

Second, the link between the empty sella and other disorders has been investigated in recent years, with a special focus on hormonal disorders. In a review of the literature (Debnath et al. 2016) individuals with an empty sella had a higher likelihood of hormonal disorder, hearing disturbances and psychiatric disorders. The defendant was obese, suffered from hearing loss and tinnitus, and claimed he heard voices. The defence consultants deemed the co‐occurrence of these different kinds of symptoms extremely informative: the presence of legally irrelevant symptoms (i.e., obesity and hearing problems, well documented in the medical history of the defendant) correlated with the empty sella made it impossible to exclude the influence of this anatomical abnormality on the psychiatric manifestations reported by the defendant (i.e., hearing voices).

**FIGURE 3:**
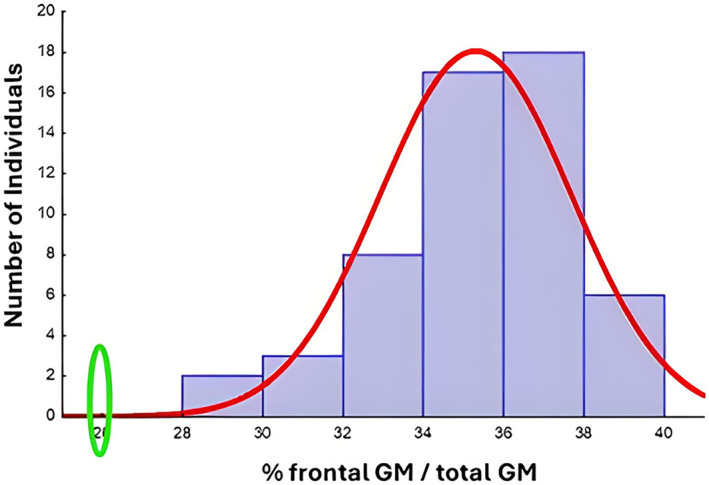
Proportion of grey matter occupied by the frontal lobe on the total grey matter. Frontal lobe volume of the defendant of case 1 (green circle) compared with the normal distribution in the population (red curve).

Third, the enlarged peri‐encephalic spaces around the frontal and parietal lobes, which were evident already at visual inspection, were analysed with quantitative techniques, leading the defence consultants to conclude that the frontal lobe of the defendant was four standard deviations smaller than that of the control group (made up of age‐matched males) (see Figure 3). A closer inspection of the brain revealed that this alteration was probably due the hypo‐development of the frontal lobe, as no signs of atrophy were present. According to authoritative literature, the volume of the frontal lobe is significantly correlated with the IQ of individuals (Duncan et al. 2000; Thompson et al. 2001). Starting from the volume of the defendant's frontal lobe his expected IQ was estimated to be 64.6, which supported the results of the neuropsychological evaluation.

**FIGURE 4:**
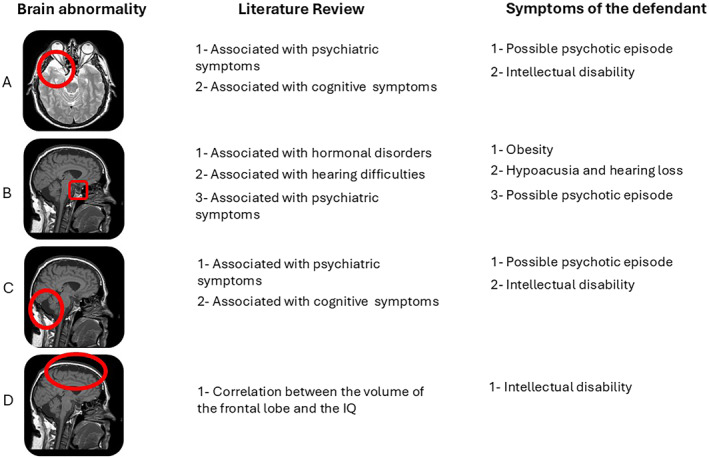
Summary of the defence reasoning. On the first column you can appreciate the ‘variant of normality’ identified in the defendant's brain. In the middle column, a summary of the findings in the literature associated with each brain abnormality. In the third column, the symptoms manifested by the defendant.

The line of reasoning followed by the defence consultants is summarised in Figure 4.

Based on the reasoning expressed above, the experts appointed by the defence claimed (at least partial) insanity for the defendant, as at the time of the crime he manifested psychotic symptoms coupled with an intellectual disability. The analysis of the crimino‐dynamic revealed that the first homicide was driven by commanding hallucinatory voices. This homicide was described as an impulsive act that he was not able to refrain. According to the INUS model of causation (Anckarsäter et al. 2009), these symptoms, combined, could have potentially led to the commission of the crime: due to his low IQ and cognitive deficits, he was not able to rationally compensate for the psychiatric manifestations he was experiencing for the first time (i.e., the commanding hallucinatory voices). The murder of the mother‐in‐law could be potentially explained by his cognitive symptoms and in particular his diminished capacity to see alternative responses (defective counterfactual thinking) and by the presence of an abnormal way of reasoning. In particular, the defendant realised the woman could not live without her daughter and the only solution he found was to kill her. A peculiar way of reasoning, which did not contemplate the recognition of absurdities, was also observed during the clinical interviews, where he complained about being in a cell with ‘criminals’, not considering that the crime he committed was much more severe than the one committed by his cellmates.

#### 4.1.4 Have Neuroimaging Data Been Over‐Interpreted in This Case?

To understand whether brain imaging data were overinterpreted, we applied the guidelines described above (Scarpazza et al. 2018). Their principles were not violated: (i) neuroimaging was used in the presence of clinical symptoms; (ii) the criminal behaviour was not considered as a symptom to put in anatomo‐clinical correlation with the brain abnormalities; (iii) the presence of insanity, psychosis, and intellectual disability was not inferred from the brain data. To further confirm the correct application of structural neuroimaging, the reasoning of the defence consultants is explained following the questions in the guidelines (Scarpazza et al. 2018):

- Is there a descriptive diagnosis? Yes. The defendant manifested a low IQ, an impaired performance at the cognitive evaluation, possibly a first episode of psychosis at the time of the crime, obesity, and hearing problems.
- Is this descriptive diagnosis potentially related to the commission of the crime? Yes, as already detailed above.
- Does neuroimaging reveal significant findings? Yes, in the form of four different brain abnormalities.
- Are these abnormalities in anatomo‐clinical correlation with the descriptive diagnosis? Yes. The brain abnormalities (of which the defendant was not aware) were correlated to the symptoms reported by the defendant. This holds true for the clinical manifestations that are not legally relevant (i.e., obesity, hearing problems), which supports the idea that the brain abnormalities cannot be considered clinically silent. Therefore, there is no scientific reason to exclude that these brain alterations are relevant to insanity.

#### 4.1.5 How Was Neuroimaging Relevant in This Case?

One of the main concerns with the application of neuroimaging in insanity evaluation is a lack of added value compared to the classical approach. Conversely, the case presented here highlights how structural brain scans can be useful in a real forensic case.

As clearly described, the neuroimaging data were not used to directly support insanity (i.e., the brain defect caused the insanity), but to support the existence of clinical symptoms potentially related to insanity. The conclusions reached by the defence consultants are rooted in the clinical evidence gathered in the evaluation. Although there was no previous clinical evidence of psychiatric and cognitive symptoms, the pattern of legally irrelevant symptoms was well‐established in the clinical history of the defendant. In this context, neuroimaging data did not establish whether the defendant was sane or insane but helped in disentangling the clinically difficult situation. Indeed, four brain alterations were found in the defendant. Although each single brain alterations can be found in the healthy population (Vernooij et al. 2007), finding four clinically silent abnormalities in the same person is, by simple probability rules, extremely unlikely. Furthermore, these abnormalities were in anatomo‐clinical correlation not only with the psychiatric and cognitive symptoms of the defendant, but also with the non‐legally relevant symptoms (e.g., obesity, hearing loss, etc.), strengthening the hypothesis that they cannot be considered ‘normal variant of normality with no clinical correlates’.

#### 4.1.6 Analysis of Biases

In this case, the disagreement between the experts clearly lies at the level of the conclusions, as described by the HEP model (Dror 2016; Dror and Murrie 2018). Indeed, all of them observed the same symptoms, but interpreted them differently: the judge's expert witnesses concluded that the defendant was malingering, whereas the defence consultants believed his symptoms were genuine and that they were correlated to the commission of the crime. The same is true for brain images: all the experts agreed on the findings, but the judge's expert interpreted them as ‘normal variants of neuroanatomy’, while the defence expert claimed that it was not possible to rule out that the brain abnormalities are related to the defendant's symptomatologic presentation due to the presence of a strong anatomo‐clinical correlation.

In this case, the multidisciplinary approach was paramount to reach a scientifically solid conclusion, with the use of neuroimaging playing an important part. Data from different sources (clinical interview, neuropsychological testing, neuroimaging data) provided convergent data supporting the presence of psychiatric and cognitive symptoms, which are in turn relevant for insanity.

### 4.2 Discussion

The introduction of neuroimaging in criminal trials, though not recent, still manages to stir up debates and controversies in the scientific literature about forensic psychiatry and psychology (Rigoni et al. 2010; Scarpazza, Pellegrini, et al. 2018; Vitacco et al. 2021). One key point often criticised by opponents to the use of neuroimaging in court is the overly free interpretation that experts often give to the data, not the use of these techniques per se (Vitacco et al. 2020, 2021). A critical issue raised by scientists who express concerns regarding the introduction of imaging in criminal trials refer to the risk of overinterpreting the data, meaning that insanity is inferred just from the presence of a brain alteration without the support of any behavioural data. There are a few reported cases in the literature, in which attorneys used the presence of a brain abnormality alone to claim their clients were insane when they committed a crime. The problem of over‐interpretation might seem the most pressing one in forensic psychiatry and psychology, as an over‐interpretation of brain scans might lead to an unjustified NGRI verdict. In turn, this would imply that someone who committed a crime while capable of understanding the disvalue of their actions and of choosing to do otherwise would not receive the sentence they deserve. On the contrary, failing to recognize a defendant's insanity, be it partial or total, might not seem as serious when considering the problem from a public safety perspective. Nonetheless, most jurisdictions introduced the insanity defence to safeguard people who may commit crimes as they are incapable of understanding the disvalue of such actions or of selecting a behavioural alternative (Adjorlolo et al. 2019; Desmarais et al. 2008). We therefore believe that drawing attention to the problem of under‐interpretation of brain imaging is of key importance, because ignoring it would be going against the very reason for which insanity evaluations were established in the first place. Moreover, as forensic neuropsychologists and neuroscientists strive for a more scientific approach to insanity evaluations (Scarpazza, Miolla, et al. 2021; Scarpazza, Zampieri, et al. 2021), it is highly important to ensure that every step of the evaluation is conducted by using the correct methodology. In the case of structural neuroimaging, this implies correctly interpreting any significant results that may emerge, always keeping in mind that the criteria for legal insanity are first and foremost behavioural (Scarpazza et al. 2018, 2021). In fact, brain abnormalities and defects can happen to be not relevant for insanity, especially when there is no anatomo‐clinical correlation between the brain alteration and the behavioural symptoms, as in the cases of Weinstein and Stevanin detailed within the introduction. It is therefore of utmost importance to provide correct and valid argumentations for the presence or absence of said correlations by taking into consideration all available data. In our opinion, the under‐interpretation of brain data is as dangerous as their over‐interpretation, as in both cases the consequence can be an incorrect sentence.

Some authors believe that neuroimaging adds no value to insanity evaluations, but we strongly believe its value is relevant in the context of a multidisciplinary evaluation. Insanity evaluations are considered scientific evidence that should help the judge to reach a decision ‘beyond any reasonable doubt’. In our opinion, the error rate of the traditional approach to insanity evaluations is too high to provide scientific evidence with the degree of certainty required in criminal trials. Scientific research on the topic revealed that the inter‐rater reliability on insanity evaluations is low (Guarnera and Murrie 2017), even in jurisdictions in which the evaluation process is not adversarial (Gowensmith et al. 2013). Gowensmith et al. (2013) found a 50% agreement between three independent experts summoned by the judge on a simple case (i.e., the defendant suffered from a full‐blown psychotic attack, or was hospitalised in a psychiatric facility short before committing the crime). This low inter‐rater reliability makes it imperative to find a way to mitigate this problem. In this context, the multidisciplinary approach (Scarpazza, Zampieri, et al. 2021) does not aim to replace but rather to integrate the standard methodology applied in insanity evaluations. Neuroimaging is a non‐necessary but at times important component of this approach. While brain scans do not directly provide information about insanity (i.e., there are no brain regions related to insanity, as it is a strictly juridical rather than scientific construct and can therefore be linked to different pathologies; but see Darby, Joutsa, et al. (2018) for preliminary data on the neural basis of free will), they may reduce uncertainty regarding the clinical status of the defendant providing support and/or clarifications on the cognitive and psychopathological symptoms. We suggest that neuroimaging data should be considered together with other clinical records and interpreted within the clinical picture of the defendant. This is critical to reduce biases at the level of the observations and the level of the conclusion of the HEP model (Dror 2016; Dror and Murrie 2018), and allowing disagreement at the level of the impact of mental health status on the ability to understand and to will. In Italy some judges are starting to ask expert witnesses to describe the clinical condition of the defendant without reaching a conclusion on insanity as the ultimate decision on insanity is left to the judge. Judges will be thus provided with the clinical information necessary to reach a decision beyond any reasonable doubt.

In conclusion, we believe that many limitations of insanity evaluations may be solved with an integrated and scientific approach, in which neuroimaging might be of use to test a scientific hypothesis. In our opinion, the scientific community should focus on avoiding both over‐ and under‐interpretation of neuroimaging data, as both these mistakes can lead to unjust sentencing. It is not possible to dismiss neuroimaging a priori, as in some complex cases it might be useful to acquire neuroimaging data in the context of insanity evaluations. In this paper, we focused on the issue of potential under‐interpretation of results, as it is a topic still unexplored in literature, but other papers described cases in which neuroimaging results were not useful for the insanity evaluation (e.g., the Stevanin case), suggesting caution in interpreting neuroimaging results and showing the mistakes that may emerge from wrong interpretation (Scarpazza et al. 2018).

This paper represents the opinion of the authors that capitalizes on the description of a case to highlight the importance of avoiding under‐interpretation of brain imaging data. To date, empirical data supporting or refuting this opinion is still lacking. In the future, the scientific community should conduct empirical research on this topic, as other authors already did for the usefulness of behavioural genetic (Fuss et al. 2015).

## Author Contributions

**Camilla Frangi:** conceptualization, writing – original draft. **Alexa Schincariol:** conceptualization, writing – original draft. **Pietro Pietrini:** conceptualization, supervision, writing – review and editing. **Giuseppe Sartori:** conceptualizazion, supervision, writing – review and editing. **Stefano Ferracuti:** conceptualization, supervision, writing – review and editing. **Cristina Scarpazza:** conceptualization, supervision, writing – review and editing.

## Ethics Statement

The defendant provided informed consent to the use of the data described here for research purposes. All data were anonymized.

## Conflicts of Interest

The authors declare no conflicts of interest.

## Data Availability

The data that support the findings of this study are available on request from the corresponding author. The data are not publicly available due to privacy or ethical restrictions.

## Endnotes

^1^
Daubert v. Merrell Dow Pharmaceuticals Inc., 509 U.S. 579 (1993).

^2^
People v. Weinstein, 591 N.Y.S.2d 715 N.Y. Sup. Ct. 1992.

^3^
Art. 85 of the Italian Penal Code. ‘No one can be punished for an act considered as a crime if, at the moment it was committed, they were not criminally responsible. A person is criminally responsible if they have the capacity to understand and to will’.

^4^
Art. 88 of the Italian Penal Code. ‘One is not criminally responsible if, at the moment the act was committed, they were, due to illness, in such a state of mind as to exclude the ability to understand or to will’.

^5^
Art. 89 of the Italian Penal Code. ‘Anyone who, at the moment they committed the act, was, due to illness, in such a state of mind as to greatly diminish, without excluding, their capacity to understand or will, is responsible for the crime committed; however, the sentence is reduced’.
